# Non-*cyp51A* Azole-Resistant *Aspergillus fumigatus* Isolates with Mutation in HMG-CoA Reductase 

**DOI:** 10.3201/eid2410.180730

**Published:** 2018-10

**Authors:** Daisuke Hagiwara, Teppei Arai, Hiroki Takahashi, Yoko Kusuya, Akira Watanabe, Katsuhiko Kamei

**Affiliations:** University of Tsukuba, Tsukuba, Japan (D. Hagiwara);; Chiba University, Chiba City, Japan (D. Hagiwara, T. Arai, H. Takahashi, Y. Kusuya, A. Watanabe, K. Kamei)

**Keywords:** azole resistance, drug resistance, *Aspergillus fumigatus*, non-*cyp51A*, HMG-CoA reductase, Hmg1, fungi, Japan, antimicrobial resistance, mutation

## Abstract

The recent increase in azole-resistant *Aspergillus fumigatus* is a global concern. Identifying the mutations that confer azole resistance is essential for developing novel methods for prompt diagnosis and effective drug treatment. We screened *A. fumigatus* clinical isolates for novel mutations conferring azole resistance. We compared the genomic sequences of susceptible and resistant isolates without mutations in *cyp51A* (non-*cyp51A*) and found mutations in *hmg1* and *erg6* involved in ergosterol biosynthesis. We also found the novel mutations in these genes in azole-resistant isolates with different genetic backgrounds. The resistant isolates with mutations in *hmg1* showed increased intracellular ergosterol levels compared with susceptible isolates. This finding supports the concept that the ergosterol level is a determinant for resistance to any class of azoles. Multiple isolates with increased resistance to azole possessed a mutation in *hmg1*, indicating that this mutation is widely present in non-*cyp51A* azole-resistant *A. fumigatus*.

*Aspergillus fumigatus* is known as an opportunistic pathogen of fungal infection and causes a high mortality rate among immunosuppressed patients (*1**,**2*). Azole drugs are considered the first-line therapy in various diseases of aspergillosis. The recent increase of azole drug-resistant *A. fumigatus* is a major limitation of therapeutic strategies in clinical settings and a concern throughout the world (*3*). Most azole-resistant strains harbor a mutation in the *cyp51A* gene, encoding a target protein for azole drugs (*4*). The point-mutated Cyp51A has lower binding affinity to azole drugs, causing azole resistance in these strains (*5*). The amino acid substitution in Cyp51A protein is thought to occur during azole therapy inside hosts. In addition, 34 bp or 46 bp of tandem repeats in the promoter region of *cyp51A* cause azole resistance (*6*). The high incidence of such tandem repeat–type resistant strains is alarming (*7*), and it is widely accepted that the tandem repeat–type resistant mechanism is derived from exposure to azole fungicides in the environment (*8*). Whereas such *cyp51A*-related azole-resistant strains have been reported frequently in the past 10 years, strains without any mutation in the *cyp51A* gene showing low susceptibility to azole drugs have, to some extent, been isolated (*9*). Which mutation leads to azole resistance has thus far been determined in only a few non-*cyp51A* azole-resistant strains. For a deeper and broader understanding of the molecular mechanisms underlying resistance to antifungal drugs, the non-*cyp51A* azole-resistant strains have become the focus of attention.

The *cdr1B* gene, encoding an ABC transporter, plays a role in azole resistance. Deletion of the gene resulted in azole-sensitive phenotypes (*10*), and an azole-resistant strain with constitutive expression of *cdr1B* has been isolated from a patient (*11*). The expression of *cdr1B* is dependent on AtrR, a recently characterized transcriptional factor (*12*), but the reason for the constitutive expression remains unclear. A strain showing azole resistance with a mutation (P88L) in the *hapE* gene has also been isolated; the mutation was discovered by genome comparison between azole-resistant strains and corresponding susceptible strains (*13*). A recent study showed that HapE forms a CCAAT binding complex, which plays a role in the negative regulation of *cyp51A* expression. The amino acid substitution in HapE (P88L) resulted in increased *cyp51A* expression and consequent azole resistance (*14*). 

These studies provided convincing evidence of the roles of Cdr1B and HapE in azole adaptation in *A. fumigatus*. However, each of these mutations has been reported only once in clinical isolates. Furthermore, a large portion of the non-*cyp51A* azole-resistant *A. fumigatus* strains have yet to be investigated to determine which genes and mutations could be responsible for azole resistance. To fill a gap in our understanding of the mechanism for azole resistance in the non-*cyp51A* strains, we searched for mutations in a series of isolates derived from a single patient in Japan who had chronic pulmonary aspergillosis (CPA) and isolates from other patients with and without azole resistance. 

## Materials and Methods

### *A. fumigatus* Strains

All clinical isolates used in this study were preserved at Chiba University Medical Mycology Research Center (Chiba City, Japan). We obtained 19 isolates from a patient at hospital C who had CPA (Tables 1, 2), 3 isolates (IFM 63768, IFM 63666, and IFM 63772) from another CPA patient at hospital C on different days, and additional isolates from 3 other patients with CPA at different hospitals (IFM 62140 at hospital K, IFM 64258 at hospital I, and IFM 64303 at hospital T). We also obtained 16 azole-susceptible clinical isolates (Table 3) from respiratory samples (sputa, pleural effusion, lung tissues, or bronchoalveolar fluids) or pus from a subcutaneous abscess from patients with aspergillosis in various hospitals in Japan.

**Table 1:** Number of short tandem repeats in *Aspergillus fumigatus* isolates from a single patient in Japan*

Isolate IFM no.	Date isolated (isolate set)	Short tandem repeats								
		2A	2B	2C	3A	3B	3C	4A	4B	4C
60814	2011 Sep (1st)	21	20	16	39	13	24	10	12	8
62916	2014 Sep (2nd)	21	20	16	39	13	24	10	12	8
63240	2014 Nov (3rd)	21	20	16	39	13	25	10	12	8
63241		21	20	16	39	13	25	10	12	8
63242		21	20	16	39	13	25	10	12	8
63243		21	20	16	39	13	25	10	12	8
63248	2015 Jan (4th)	21	20	16	39	13	24	10	12	8
63249		21	20	16	39	13	25	10	12	8
63537	2015 Jul (5th)	21	20	16	39	13	24	10	12	8
63594	2015 Sep (6th)	21	20	16	39	13	24	10	12	8
63595		21	20	16	39	13	24	10	12	8
63596		21	20	16	39	13	24	10	12	8
63711	2015 Dec (7th)	21	20	16	39	13	24	10	12	8
63712		21	20	16	39	13	24	10	12	8
63713		21	20	16	39	13	24	10	12	8
63714		21	20	16	39	13	24	10	12	8
64138	2016 Apr (8th)	21	20	16	39	13	24	10	12	8
64139		21	20	16	39	13	24	10	12	8
64173	2016 May (9th)	21	20	16	39	13	24	10	12	8

*IFM, Institute of Food Microbiology (now Medical Mycology Research Center), Chiba University, Chiba City, Japan.

**Table 2:** Characteristics of *Aspergillus fumigatus* isolates from a single patient in Japan*

Isolate IFM no.	Date isolated (isolate set)	MCFG MEC, mg/L	MIC, mg/L					Gene with mutation		
			AMPH	ITCZ	VRCZ	PSCZ		*Cyp51*	*Hmg1*	*Erg6*
60814	2001 Sep (1st)	<0.015	0.5	0.5	0.5	1		–	–	–
62916	2014 Sep (2nd)	<0.015	0.5	>8	>8	8		G448S	S269F	E49K
63240	2014 Nov (3rd)	<0.015	1	>8	>8	4		–	S269F	A350T
63241		<0.015	0.5	>8	>8	8		–	S269F	A350T
63242		<0.015	1	>8	>8	8		–	S269F	A350T
63243		<0.015	1	>8	>8	8		–	S269F	A350T
63248	Jan 2015 (4th)	ND	ND	ND	ND	ND		–	S269F	E49K
63249		<0.015	1	4	8	8		G448S	S269F	E49K
63537	2015 Jul (5th)	<0.015	1	>8	8	8		–	S269F	A350T
63594	2015 Sep (6th)	<0.015	1	>8	>8	8		–	S269F	A350T
63595		<0.015	1	>8	>8	>8		G448S	S269F	E49K
63596		<0.015	1	>8	>8	8		–	S269F	A350T
63711	2015 Dec (7th)	ND	ND	ND	ND	ND		–	S269F	E49K
63712		ND	ND	ND	ND	ND		G448S	S269F	E49K
63713		ND	ND	ND	ND	ND		G448S	S269F	E49K
63714		<0.015	1	>8	8	4		–	S269F	E49K
64138	2016 Apr (8th)	ND	ND	ND	ND	ND		–	S269F	E49K
64139		ND	ND	ND	ND	ND		G448S	S269F	E49K
64173	2016 May (9th)	<0.015	0.5	>8	8	4		–	S269F	A350T

*AMPH, amphotericin B; IFM, Institute of Food Microbiology (now Medical Mycology Research Center), Chiba University, Chiba City, Japan; ITCZ, itraconazole; MCFG, micafungin; MEC, minimal effective concentration; ND, not determined; PSCZ, posaconazole; VRCZ, voriconazole; –, no mutation.

**Table 3:** Characteristics of azole-susceptible isolates of Aspergillus fumigatus in Japan*

Isolate IFM no.	MCFG MEC, mg/L	MIC, mg/L				Gene with mutation			Short tandem repeats					
		AMPH	ITCZ	VRCZ		*hmg1*	*erg6*		3A	3B	3C	4A	4B	4C
49435	<0.015	1	0.25	0.25		–	–		52	17	20	10	11	11
50268	<0.015	0.5	0.25	0.125		–	–		14	10	11	13	9	10
50669	<0.015	1	0.5	0.25		–	–		12	18	20	16	9	5
50999	<0.015	1	0.5	0.5		–	–		33	12	37	11	9	5
51748	<0.015	0.5	0.125	0.125		–	–		26	11	29	25	10	8
51977	<0.015	1	0.25	0.25		–	–		25	9	7	12	11	17
57130	<0.015	1	0.25	0.125		–	–		47	21	19	10	11	5
58402	<0.015	1	0.5	0.5		–	–		27	10	5	9	9	5
60065	<0.015	1	1	0.5		–	–		15	17	17	17	16	10
60516	<0.015	1	1	1		–	–		22	11	7	16	11	8
60901	<0.015	1	0.5	0.5		H564Y	–		23	28	21	8	11	10
61572	<0.015	1	0.5	0.5		–	–		59	19	7	10	14	8
61960	<0.015	1	0.5	0.5		–	–		27	11	35	10	9	10
62674	<0.015	1	1	2		–	–		35	10	14	16	10	10
62709	<0.015	1	0.5	0.5		–	–		38	18	7	10	14	9
62821	<0.015	1	0.5	0.25		–	–		37	14	7	10	11	10

*AMPH, amphotericin B; IFM, Institute of Food Microbiology (now Medical Mycology Research Center), Chiba University, Chiba City, Japan; ITCZ, itraconazole; MCFG, Micafungin; MEC, minimal effective concentration; VRCZ, voriconazole; –, no mutation.

### Microsatellite Genotyping

For genotyping, we PCR amplified and sequenced 6 loci of ≈400 bp from all of the isolates as described previously (*15*). We counted the repeat numbers of each region (2A, 2B, 2C, 3A, 3B, 3C, 4A, 4B, and 4C) from the sequence.

### Antifungal Susceptibility Testing

We performed antifungal susceptibility testing as described previously (*16*). In brief, we performed tests in triplicate using amphotericin B (AMPH), itraconazole (ITCZ), voriconazole (VRCZ), and posaconazole (PSCZ) in RPMI 1640 medium (pH 7.0) at 35°C, according to the Clinical and Laboratory Standards Institute reference method for broth microdilution (https://clsi.org/standards/products/microbiology/documents/m38), with partial modifications using the dried plate for antifungal susceptibility testing (Eiken Chemicals, Tokyo, Japan).

### Sequencing *cyp51A*, *erg6*, and *hmg1* Genes

To detect mutations in *cyp51A*, *erg6*, and *hmg1* genes, we performed PCR amplification and sequenced these regions using appropriately designed primers (Technical Appendix Table 1). We performed sequence variant detection by comparison with reference sequences from GenBank and Fungi DB (http://fungidb.org/fungidb/) (GenBank accession no. AF338659 for *cyp51A*, Fungi DB accession nos. AFUB_020770 for *hmg1,* and AFUB_099400 for *erg6*).

### Whole-Genome Sequencing

We performed whole-genome sequencing by using next-generation methods as described previously (*15*). In brief, we extracted genome DNA from overnight-cultured mycelia by DNeasy Plant Mini Kit (QIAGEN, Hilden, Germany) or by phenol-chloroform extraction and Nucleobond AXG column (TaKaRa, Kusatsu, Japan) with Nucleobond Buffer Set III (TaKaRa). For analyzing IFM 60814 and IFM 63240, we prepared a fragmented DNA library by using a Nextera DNA sample preparation kit according to the manufacturer’s instructions (Illumina, San Diego, CA). The mean length of the libraries was 376–674 bp. We performed sequencing in a paired-end 2 × 150 bp mode on a MiSeq system (Illumina). For analyzing IFM 63666 and IFM 63768, we prepared a fragmented DNA library using KAPA HyperPlus Library Preparation Kit (Kapa Biosystems, Wilmington, MA) according to the manufacturer’s instructions. We performed sequencing in a paired-end 2 × 300 bp mode on a MiSeq system (Illumina).

### Single-Nucleotide Variant Detection

To search for single-nucleotide polymorphisms (SNPs) between IFM 60814, the first isolate obtained, and IFM 63240–63243, the third set of isolates, we performed read-mapping and SNP detection using CLC Genomics Workbench (CLC bio, Aarhus, Denmark). In brief, we trimmed the reads from each isolate and mapped them to the Af293 reference genome. By comparing them with the Af293 sequence, we detected sites with SNPs in IFM 60814, IFM 63240, IFM 63241, IFM 63242, and IFM 63243. We also compared the sets of varied sites between IFM 60814 and IFM 63240–63243. We considered the unique sites common in IFM 63240–63243 to be the SNPs between the first isolate and the third set of isolates.

We cleaned the read datasets of IFM 63666 and IFM 63768 using Trimmomatic version 0.33 (*17*). We trimmed reads to generate de novo assembly of the draft genome of IFM 63666 using Platanus version 1.2. 4 with default parameters (*18*). We performed annotation of all predicted open reading frames of the draft genome using AUGUSTUS version 2.5.5 (*19*). For nucleotide differences between IFM 63666 and IFM 63768, we mapped the trimmed reads of IFM 63768 to the draft genome of IFM 63666 using SMALT version 0.7.6 (http://www.sanger.ac.uk/science/tools/smalt-0). We identified SNPs using SAMtools version 0.1.19–44428cd (*20*) and filtered with >10-fold coverage, >30 mapping quality, and 75% consensus using in-house scripts (*21**,**22*). We annotated the functional effect of SNPs with SnpEff version 4.1l (*23*).

### Growth Test for Ergosterol-Related Inhibitors

We performed disk diffusion assay using the protocol described by Qiao et al. (*24*). We spread a 200-µL suspension (1 × 10^6^ conidia/mL) of each of the tested isolates uniformly onto YGA (glucose 2%, yeast extract 0.5%, agar 2%, trace element). We impregnated blank paper disks 8 mm in diameter with 2.5 µg amphotericin B, 100 µg nystatin, or 40 µg lovastatin and placed the disks onto the center of the inoculated agar plates. We incubated the plates at 37°C and measured the diameters of the zones of inhibition 48 hours later. We performed each assay 3 times and calculated the mean diameter.

### Ergosterol Quantification

We conducted total ergosterol extraction using the protocol described by Arthington-Skaggs et al. and Alcazar-Fuolietal (*25**,**26*). We cultured *A. fumigatus* isolates for 20 h in glucose minimal medium. We harvested mycelia by filtration, washed them with sterile water, and then dried and weighed them. We added 3 mL of 25% alcoholic potassium hydroxide solution to dried mycelia and mixed for 1 min, then incubated the mixture in an 80°C water bath for 1 h. After incubation, we added water and 3 mL of pentane and mixed for 3 min. We transferred the upper pentane layer to a clean glass tube for evaporation. We dissolved the dried samples in 1 mL of methanol and filtered it through a 0.22-µm pore size membrane filter. We analyzed ergosterol content using the Shimadzu LC-20A system (Shimadzu, Kyoto, Japan) with COSMOSIL 5C^18^-MS-II column (4.6 mm ID × 100 mm; Nacalai Tesque, Kyoto, Japan). We established a flow rate of 1 mL/min of acetonitrile to water (95:5 vol/vol). We used peak areas and heights recorded at a 254-nm wavelength in an RF-20Axs (Shimadzu) for quantification, expressing total ergosterol concentration as µg ergosterol per mg fungal dry weight. We repeated each experiment >3 times.

### Construction of Mutant Erg6, Hmg1, and Cyp51A Expressed Strains

To construct transformants expressing *erg6*, *hmg1*, or *cyp51A* with the mutation, we cloned the alleles using the shuttle vector pPTR I (Takara Bio, Otsu, Japan) and GeneArt Seamless Cloning and Assembly Kit (Invitrogen, Tokyo, Japan). Primers used for the cloning are listed in Technical Appendix Table 2. We used genome DNAs of IFM 60814 (for wild-type [WT] gene) and IFM 63240 (for mutated allele) as templates to clone the alleles. The resultant plasmids were pPTRI-*erg6*^WT^, pPTRI-*hmg1*^WT^, pPTRI-*cyp51A*^WT^, pPTRI-*erg6*^A350T^, pPTRI-*hmg1*^S269F^, and pPTRI-*cyp51A*^G448S^, which we used for transformation of isolate IFM 60814. After selection with pyrithiamine, we verified the sequences of each gene of the candidate transformants by Sanger sequencing.

## Results

### Multiazole Resistant Strains from a Single Patient

We recovered a total of 19 *A. fumigatus* isolates from 1 patient on 9 testing dates during September 2011–May 2016 (Tables 1, 2). Microsatellite analysis showed almost identical short tandem repeats across the isolates, derived from a genetically clonal background. The patient started treatment with VRCZ after the first isolation of *A. fumigatus* in September 2011. Whereas the first isolate (IFM 60814) was susceptible to antifungal drugs including ITCZ, VRCZ, and PSCZ, later isolates showed resistance to the azoles (MICs: ITCZ, 4 to >8; VRCZ, 8 to >8; PSCZ, 4 to >8). By sequencing the *cyp51A* gene, we found amino acid substitution G448S IFM 62916 (second isolation date), IFM63248 (fourth isolation date), IFM63595 (sixth isolation date), IFM63712 (seventh isolation date), IFM63713 (seventh isolation date), and IFM64139 (eighth isolation date). These isolates were most likely resistant to VRCZ as a result of the G448S mutation, which has been reported to confer VRCZ resistance in *A. fumigatus*. However, the multiazole resistance mechanism in the isolates with no G448S mutation remained unknown. The growth of isolate IFM 62916 was markedly impaired with glucose minimal medium and potato dextrose agar, whereas the isolates from the third isolation date also showed a moderately delayed growth compared with those of the first strain (Technical Appendix Figure 1).

### Genome-Wide Sequence Comparison of Azole-Susceptible and Azole-Resistant Isolates

To gain insight into the multiazole resistance mechanism in the non-*cyp51A* strains, we sequenced the genomes of the isolates from the first (IFM 60814) and third (IFM 63240–63243) testing dates by next-generation sequencing. Compared with IFM 60814, IFM 63240–63243 commonly showed 7 nonsynonymous mutations, including 6 SNPs (4 amino acid substitutions and 2 nonsense mutations, which generate termination codon) and a 1-bp deletion resulting in a frame shift of the protein (Technical Appendix Table 2). The 6 genes with mutations included *erg6*, encoding sterol 24-C-methyltransferase and *hmg1*, encoding hydroxymethylglutaryl-CoA (HMG-CoA) reductase. The mutation of *erg6* (A350T) resided in a Sterol_MT_C domain (PF08498) in the C-terminus, whereas the mutation of *hmg1* (S269F) was located at the beginning of the sterol-sensing domain (PF12349) (Technical Appendix Figure 2). Because both of these genes are functionally related to the ergosterol biosynthesis pathway, we assumed that these mutations affect ergosterol biosynthesis and the consequential azole resistance in the strains.

We examined the rest of the isolates from the patient for mutations in the *hmg1* and *erg6* genes. Despite the presence of A350T, we identified E49K in the *erg6* gene in several isolates, whereas Hmg1 S269F existed in all isolates tested (Tables 1, 2). These results indicate that all of the isolates showing multiazole resistance had mutations in both *hmg1* and *erg6* genes, regardless of the mutation in *cyp51A* gene.

### Different Ergosterol Levels in Multiazole-Resistant Isolates

**Figure 1:**
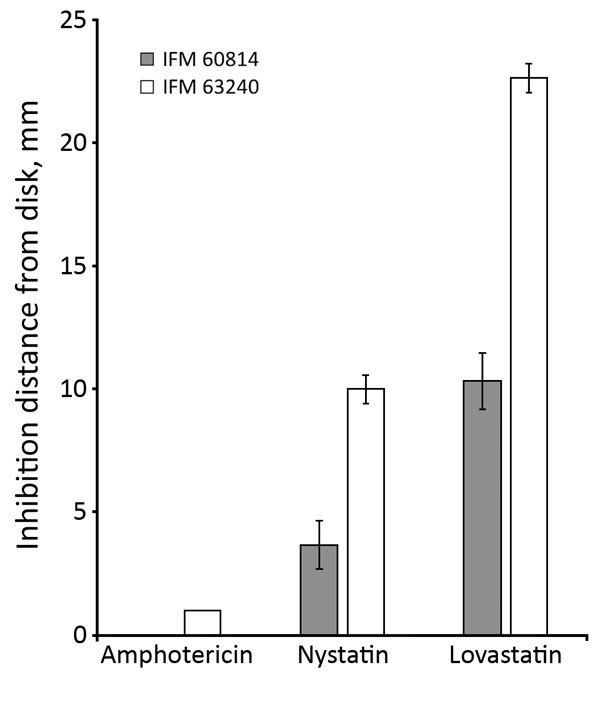
Growth inhibition by lovastatin and polyene drugs in azole-susceptible and azole-resistant *Aspergillus fumigatus* isolates from a patient in Japan. Growth inhibition tests were conducted by disk assay. IFM 60814 is a susceptible isolate identified on the first date of testing; IFM 63240 is a resistant isolate identified on the third date of testing, with mutations in *hmg1*. The results of repeated experiments are expressed as mean ± SD (error bars).

**Figure 2:**
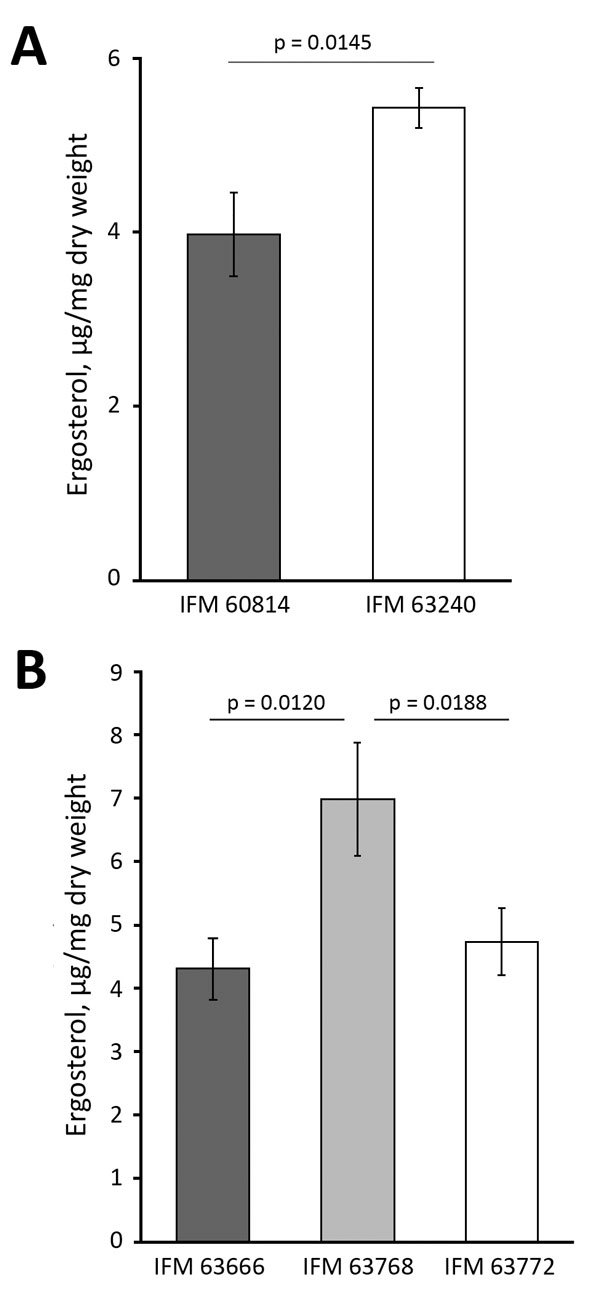
Total ergosterol content of azole-susceptible and azole-resistant *Aspergillus fumigatus* isolates in Japan. Ergosterol was quantified in the sets of IFM 60814, a susceptible isolate identified on the first date of testing, and IFM 63420, a resistant isolate identified on the third date of testing, both from 1 patient (A); and IFM 63666, IFM 63768, and IFM 63772, all isolated from other patients in the same hospital (B). The results of repeated experiments are expressed as mean ± SD (error bars). The significance of total ergosterol content was determined by the Student *t*-test (unpaired, unequal variance). A p value <0.05 was considered significant.

We investigated the phenotypes associated with the ergosterol biosynthesis pathway in IFM 63240, a representative of the isolates from the third testing day. Disk diffusion assay showed that this isolate was more sensitive to polyene drugs, amphotericin B, and nystatin, suggesting that ergosterol production might be differentially regulated (Figure 1). The ergosterol content in the cells, measured by HPLC, showed significant increase (p = 0.0145) in the third set of isolates compared with the first isolate (Figure 2, panel A). At the same time, we found no differences between the first and third isolates regarding sensitivity to terbinafine, which interferes with the early stage of ergosterol biosynthesis, and fenpropimorph, which inhibits ergosterol biosynthesis (data not shown). Of note, the isolate from the third set showed increased sensitivity to lovastatin, an inhibitor of HMG-CoA reductase (Figure 1).

### Evaluation of Mutant *erg6* and *hmg1* by Transformation

Considering the different level of ergosterol biosynthesis in the third set of isolates, we wanted to determine whether the mutation in *erg6* and/or *hmg1* genes was responsible for the phenotypes (i.e., sensitivity to polyenes and resistance to azoles). To address this question, we constructed the isolates carrying these mutant alleles as well as the corresponding wild-type gene by introducing *erg6*^A350T^ or *hmg1*^S269F^ into the first strain (hereafter designated as 1st+*erg6*^A350T^ and 1st+*hmg1*^S269F^). These mutant alleles were ectopically expressed in the transformed strains. We also constructed the strain carrying *cyp51A*^G448S^ (1st+*cyp51A*^G448S^) for verification of the role of the mutation in azole resistance as previously reported (*27*). The MICs for azoles were determined in these strains (Table 4), showing that introducing *erg6*^A350T^ or *hmg1*^S269F^ did not affect susceptibility to azoles, whereas the *cyp51A*^G448S^ conferred resistance to VRCZ.

**Table 4:** Antifungal susceptibility test of mutants of *erg6* and *hmg1* from *Aspergillus fumigatus* isolates in Japan by transformation

Strain	Genotype	MCFG MEC, mg/L	MIC, mg/L				
			AMPH	ITCZ	VRCZ	MCZ	PSCZ
1st+*erg6^WT^*	*cyp51A, erg6, +erg6^WT^, hmg1*	<0.015	2	1	1–2	2	1–4
1st+*erg6^A350T^*	*cyp51A, erg6, +erg6^A350T^, hmg1*	<0.015	2	1	0.5–1	2	4
1st+*hmg1^WT^*	*cyp51A, erg6, hmg1, +hmg1^WT^*	<0.015	2	1	1	2	1–4
1st+*hmg1^S269F^*	*cyp51A, erg6, hmg1, +hmg1^S269F^*	<0.015	1–2	0.5–1	0.5	1–2	2–4
1st+*cyp51A^WT^*	*cyp51A, +cyp51A^WT^, erg6, hmg1*	<0.015	1–2	1	1–2	2–4	0.25–4
1st+*cyp51A^G448S^*	*cyp51A, +cyp51A^G448S^, erg6, hmg1*	<0.015	1–2	4	>8	4	4

*AMPH, amphotericin B; ITCZ, itraconazole; MCFG, micafungin; MEC, minimal effective concentration; MCZ, miconazole; ND, not determined; PSCZ, posaconazole; STR, short tandem repeats; VRCZ, voriconazole.

### Mutations in *erg6* and *hmg1* in Other Non-*cyp51A* Azole-Resistant Isolates

We studied whether the other non-*cyp51A* azole-resistant isolates possess mutations in *erg6* or *hmg1*. Over the past decade, we have collected clinical azole-resistant *A. fumigatus* strains in Japan: 4 non-*cyp51A* azole-resistant isolates (IFM 62140, IFM 64258, IFM 64303, and IFM 63768) showing a VRCZ MIC of >8 (Table 5). Each isolate was from a patient who had a history of treatment with VRCZ before isolation. Although IFM 64303 has no mutations in *erg6* and *hmg1* genes, the other 3 isolates possessed different mutations in *erg6* and/or *hmg1*. IFM 62140 had *erg6*^A75S^ and *hmg1*^F261_del^, IFM 63768 had *hmg1*^S269Y^, and IFM 64258 had *erg6*^V206A^ and *hmg1*^F390Y^, which were different from the pattern observed in IFM 63240. These results suggested a potential link between azole resistance and the mutations in *erg6* and *hmg1* genes. Notably, IFM 63768 and IFM 63240 had a mutation in Hmg1 at S269 in which serine was substituted by different amino acids (i.e., Hmg1^S269Y^ and Hmg1^S269F^). To investigate the possibility that such mutations are just frequently occurring polymorphisms, we sequenced *erg6* and *hmg1* in 16 azole-susceptible isolates. We did not find mutation in *erg6*, although 1 of the 16 isolates, IFM 60901, had a *hmg1*^H564Y^ mutation (Table 3). We did not find the mutations *hmg1*^S269F/Y^, *hmg1*^F261_del^, and *hmg1*^F390Y^ in the azole-susceptible isolates. These results suggest that the mutations in *erg6* and *hmg1* found in the azole-resistant isolates are not mere polymorphisms in *A. fumigatus* but can be implicated in azole resistance.

**Table 5:** Characteristics of *Aspergillus fumigatus* isolates from different patients in Japan*

Isolate IFM no.	Hospital	MCFG MEC, mg/L	MIC, mg/L					Gene with mutation				STRs					
			AMPH	ITCZ	VRCZ	PSCZ		*cyp51A*	*hmg1*	*erg6*		3A	3B	3C	4A	4B	4C
62140	K	<0.015	2	4	8	2		–	F261_del	A75S		42	11	14	13	9	8
64258	I	<0.015	1	4	8	4		–	F390Y	V206A		26	14	17	5	11	10
64303	T	<0.015	2	2	8	–		–	–	–		34	18	22	10	11	11
63666	C	<0.015	1	1	2	1		–	–	–		29	13	15	10	9	5
63768	C	<0.015	1	8	>8	8		–	S269Y	–		29	13	15	10	9	5
63772	C	<0.015	1	2	4	8		–	–	–		29	13	15	10	9	5

*AMPH, amphotericin B; IFM, Institute of Food Microbiology (now Medical Mycology Research Center), Chiba University, Chiba City, Japan; ITCZ, itraconazole; MCFG, micafungin; MEC, minimal effective concentration; PSCZ, posaconazole; STRs, short tandem repeats; VRCZ, voriconazole; –, no mutation.

### Comparing Genome Sequences of Non-*cyp51A* Azole-Resistant Isolate and Corresponding Isolates

In our collection, an azole-susceptible isolate (IFM 63666) was isolated 2 months before isolation of azole-resistant IFM 63768 (*hmg1*^S269Y^) from the same patient. The 2 isolates shared the same short tandem repeats, indicating a clonal lineage. We then sequenced the genomes of IFM 63768 and IFM 63666. Comparing the genome sequences, we discovered only 1 nonsynonymous mutation other than *hmg1*^S269Y^ in IFM 63768, which was I395M in Afu4g04290 encoding a putative histone H4 deacetylase protein. Although we could not see the effect of the mutation in Afu4g04290, this result supported the possibility that Hmg1^S269Y^ is the determinant for azole resistance in this isolate. We compared ergosterol levels in the cells between IFM63768 and IFM 63666 and found an increased amount in IFM 63768 with *hmg1*^S269Y^ (Figure 2, panel B). We isolated IFM 63772 from the same patient in addition to these isolates, and it showed moderate resistance to VRCZ, resistance to PSCZ, and no mutations in *erg6* and *hmg1* (Table 5). The amount of ergosterol in IFM 63772 was comparable to that of IFM 63666 (Figure 2, panel B).

## Discussion

The emergence of drug-resistant fungal strains has been on the rise in recent years, leading to a serious situation. Azole-resistant strains occurring during the course of therapy have been increasingly reported all over the world since the early discovery of an ITCZ-resistant strain (*5*). Some studies have shown that there are many azole-resistant *A. fumigatus* strains without mutation in *cyp51A* gene, and the molecular mechanisms underlying resistance in the strains remain unstudied (*28*–*30*). This finding indicates that many unclear molecular mechanisms related to azole resistance still exist. 

In this study, we screened novel mutations conferring resistance by comparing the genomic sequences of susceptible and resistant strains isolated from the same patient. We found mutations in *hmg1* and *erg6*, encoding enzymes Hmg1 and Erg6, respectively, which are involved in ergosterol biosynthesis. Hmg1 is a HMG-CoA reductase that catalyzes reduction of HMG-CoA to mevalonic acid and acts as a rate-limiting enzyme in ergosterol biosynthesis. *Erg6* encodes sterol methyltransferase, which catalyzes the conversion of lanosterol to eburicol in *A. fumigatus* (*26*). The mutations of *hmg1* we discovered were located in the sterol-sensing domain (SSD), responsible for the function of this enzyme. Thus, the mutation (*hmg1*^S269F^) might affect ergosterol biosynthesis efficiency. In fact, ergosterol levels in the strain bearing *hmg1*^S269F^ or *hmg1*^S269Y^ were increased, which led us to presume that such mutations altered the activity of Hmg1 and resulted in hyperaccumulation of ergosterol in the cells. Although further clarification is needed, one possibility is that larger amounts of azole drugs are required to inhibit growth of the cells with an increased level of ergosterol. Among the non-*cyp51A* azole-resistant strains we studied, all the strains bearing mutation in *hmg1* showed resistance to multiple azoles. This finding also supported the idea that the ergosterol level is a determinant for the resistance against any class of azoles.

Because Hmg1 acts as a rate-limiting enzyme, we investigated the phenotypes associated with ergosterol biosynthesis in the *hmg1* mutated strain. Polyene drugs such as amphotericin B and nystatin bind to ergosterol in the plasma membrane, leading to cell death by promoting membrane leakage. Of note, the strain with mutation in Hmg1 was more sensitive to these polyene drugs, compared with the strains without the mutation, likely because the azole-resistant strain has more ergosterol in the cell membrane, and polyene can increasingly access ergosterol and damage the membrane. We also found that the azole-resistant strain with Hmg1 mutation had increased susceptibility to lovastatin. Lovastatin is a competitive inhibitor of Hmg1, so the mutation may have increased the affinity to lovastatin. However, clarification for this will still be required in future studies.

Mutation of *hmg1* has been reported in experiments designed to produce azole-resistant strains in the laboratory (*31*). In this study, the mutation in *hmg1* was identified in clinical azole-resistant isolates. The mutation was discovered in several isolates with different genetic backgrounds, which strongly suggested the importance of the mutation in azole resistance.

To analyze functions of the amino acid substitutions in Cyp51A, Hmg1, and Erg6 identified here, mutant alleles (*cyp51A*^G448S^, *hmg1*^S269F^, and *erg6*^A350T^) were ectopically expressed in susceptible strains. Introducing mutated *cyp51A*^G448S^ conferred azole resistance in the host strain even in the presence of wild-type *cyp51A*. On the other hand, mutations in *hmg1* and *erg6* did not confer azole resistance when the wild type was present. The amino acid substitution of Hmg1 is present in SSD, which is a conserved motif of membrane proteins involved in sterol sensing and acts on feedback regulation of the enzymatic reaction (*32*). Therefore, the mutated Hmg1 may be impaired in the feedback regulation. In the strain expressing *hmg1*^S269F^, it is considered that wild-type *hmg1* can receive feedback inhibition, and thus the strain did not accumulate ergosterol and did not alter azole susceptibility. All mutations of *hmg1* found in this study are present in SSD, suggesting that mutations in SSD are important for conferring azole resistance.

In this study, we discovered novel genetic changes related to azole resistance. Several non-*cyp51*A azole-resistant clinical isolates harbor the mutation in *hmg1*, which suggested that this possible resistance mechanism is prevalent in non-*cyp51A* strains. We proposed that multiazole drug resistance is imparted by a mutation in a protein other than the target protein of azole drugs. Future elucidation of the molecular mechanism of the *hmg1* mutation will lead to a more complete understanding of the azole resistance mechanism in *A. fumigatus*.

**Technical Appendix.** Additional information about mutations in *Aspergillus fumigatus* isolates in Japan.
