# Supplementary material for: Non-cyp51A Azole-Resistant Aspergillus fumigatus Isolates with Mutation in HMG-CoA Reductase
Source: Emerg Infect Dis. 2018 Oct;24(10):1889–97. doi: 10.3201/eid2410.180730 (PMC6154143; doi:10.3201/eid2410.180730)
Supplement: Supplementary file 1 — Technical Appendix. Additional information about mutations in Aspergillus fumigatus isolates in Japan. [file 18-0730-Techapp-s1.pdf]

Article DOI: https://doi.org/10.3201/eid2410.180730

Non-*cyp51A* Azole-Resistant *Aspergillus fumigatus* Isolates with Mutation in HMG-CoA Reductase

Technical Appendix

Technical Appendix Table 1. Primers used in study of non- *cyp51A* azole-resistant *Aspergillus fumigatus*

Primer name	Sequence (5′ to 3′)
SQ(cyp51A)	
cyp51A_SQ1F	GAAGCGGTTAAGGCGGGAATAGACG
cyp51A_SQ2F	GAATAATTTACACTGTTCTCCTCTAG
cyp51A_SQ3F	GGTGCCGATGCTATGGCTTACGGCCTAC
cyp51A_SQ4F	CGGATCGGACGTGGTGTATGATTG
cyp51A_SQ5F	CTTGACGGTGACAAGGACTCTCAG
cyp51A_SQ6F	CGCGTGCTCCTTGCTTCACCTGGAG
cyp51A_M-R	GTAGATTGACCTCATGCGCGCATGAGCAGC
cyp51A-R	CAGGTTTTCGCACGAGCTTCTCC
SQ(hmg1)	
hmg1_SQ1F	CATTCCTGAGATTTCTCAGCATCGA
hmg1_SQ2F	TGGACGTCGATACTCTGCTTCA
hmg1_SQ3F	AGATCCCGGACACCTCCTCCGAT
hmg1_SQ4F	ACTGCGGCAGCGGACAACAGGGGCA
hmg1_SQ5F	AGCAACAAGACTAGCATCTTTGGGAG
hmg1_SQ6F	CGTGTCATCGAGAGTCTTCTCAAGAGT
hmg1_SQ7F	GCGGTCAAGATCCGTCGAGCTGTT
hmg1_SQ8F	GGTCGAAGCATTTTGACTGCGGC
hmg1_SQ9F	GGAAGCGTGGGTGGGTTCAACGCT
hmg1_SQ10F	CGTCTCAGCAGCCGTGAGTGCTGCT
hmg1_SQR1	ACTCTTGAGAAGACTCTCGATGACACG
hmg1_SQR2	CCATGTGTATTTCGGACAGCCAGC
SQ(erg6)	
erg6_SQ1F	GACAGTTGAGATAAAAAAGGTGTAAG
erg6_SQ2F	GATCGGCGAGATCCTCGTTGTGCA
erg6_SQ3F	TGTTCAGCCCGACCACATTGGCATC
erg6_SQ4F	TACTTACCGCGCGAGTCTCCTCT
erg6_SQR1	TACTCTCAATATTTCCACTTGGGCCA
cyp51A-F(pPTR-k)	GAATTCGAGCTCGGTACTGGGCAATTCAGGGGATGAACAAG
cyp51A-R(pPTR-p)	GCTTGCATGCCTGCATTTAGACAGGATCTGAAGTTTTC
erg6-F(pPTR-k)	GAATTCGAGCTCGGTACCTCCTTGAGCATCTGAACGTAAGG
erg6-R(pPTR-p)	GCTTGCATGCCTGCATGTTATTTTGAAGTCGAGCTGAATCC
hmg1-F(pPTR-k)	GAATTCGAGCTCGGTACTCCTGTTGCTGAGAAAAGGAGGG
hmg1-N-R	CCAGCGAGCGGCATTGAACAAGTAGCCG
hmg1-C-F	CGGCTACTTGTTCAATGCCGCTCGCTGG
hmg1-R(pPTR-p)	GCTTGCATGCCTGCATGCCAGCTGTTGACATACGACAGAAGC

Technical Appendix Table 2. Sequence variants in the third strain (IFM 63240) of *Aspergillus fumigatus*

Gene ID	Type	Amino acid change	Length of the protein, amino acids	Gene name	Annotation
AFUA_2G08170	SNV	Glu118*	396	–	Has domain with predicted role in regulation of transcription
AFUA_2G11830	Deletion	Trp292fs†	333	–	Hypothetical protein
AFUA_4G03630	SNV	Ala350Thr	377	*erg6*	Sterol 24-C-methyltransferase with a predicted role in glycosylphosphatidylinositol (GPI)-anchor biosynthesis
AFUA_5G05850	SNV	Trp177*	465	–	Hypothetical protein
AFUA_5G12650	SNV	Asn195Asp	499	–	Hypothetical protein
AFUA_2G03700	SNV	Ser269Phe	1130	*hmg1*	Hydroxymethylglutaryl-CoA (HMG-CoA) reductase
AFUA_5G14660	SNV	Gln99Arg	529	–	GABA permease? amino acid transmembrane transporter?

*Nonsense change, which generated termination codon
†fs, frame shift

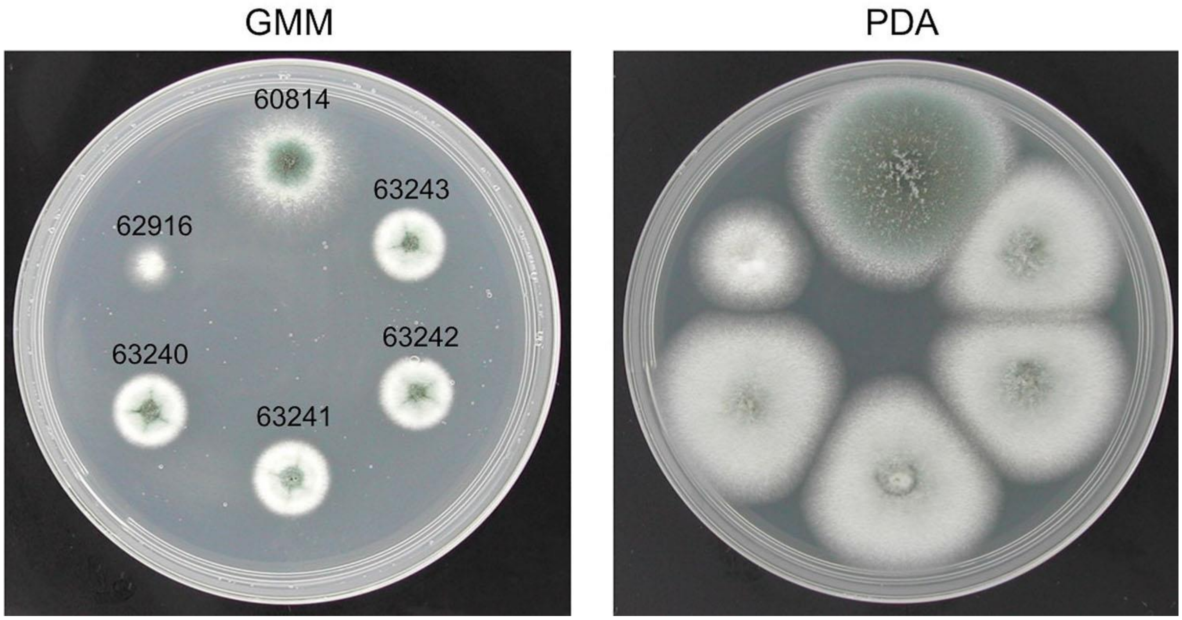

Technical Appendix Figure 1. Observed colony morphology of *Aspergillus fumigatus* strains isolated from a single patient. GMM and PD agar plates were incubated at 37°C for 48 h.

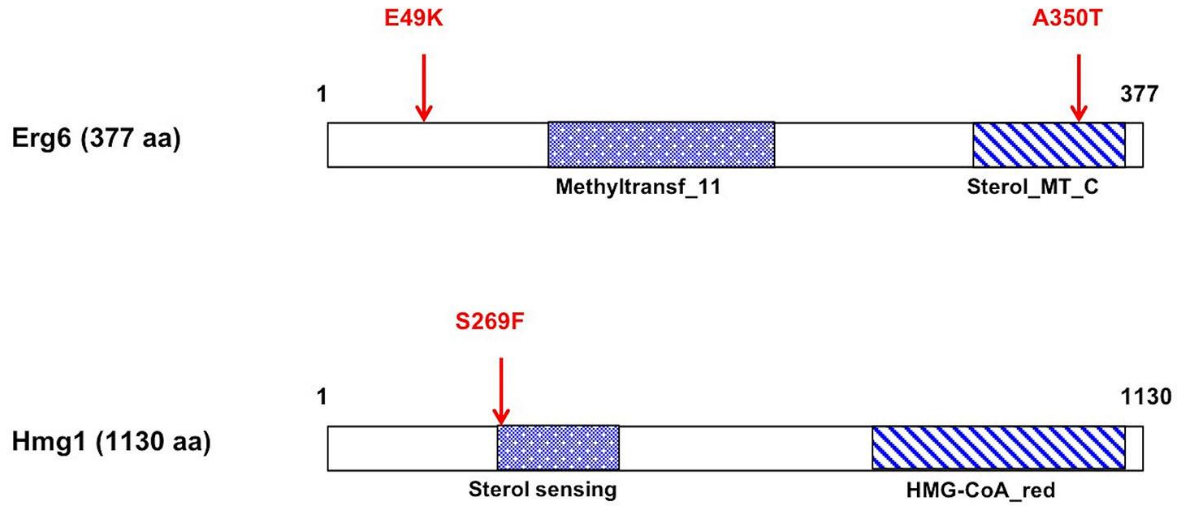

Technical Appendix Figure 2. Mutation sites of Hmg1 and Erg6. Arrows indicate mutation sites. Methyltransf_11, methyltransferase domain; Sterol_MT_C, sterol methyltransferase C-terminal domain; Sterol sensing, sterol sensing domain; HMG-CoA_red, HMG-CoA reductase domain.
